# The Effect of Valine on the Synthesis of α-Casein in MAC-T Cells and the Expression and Phosphorylation of Genes Related to the mTOR Signaling Pathway

**DOI:** 10.3390/ijms26073179

**Published:** 2025-03-29

**Authors:** Min Yang, Xinyu Zhang, Yu Ding, Liang Yang, Wanping Ren, Yu Gao, Kangyu Yao, Yuxin Zhou, Wei Shao

**Affiliations:** Xinjiang Key Laboratory of Meat and Milk Production Herbivore Nutrition, College of Animal Science, Xinjiang Agricultural University, Urumqi 830052, China; y1375724150@163.com (M.Y.); 18663955139@163.com (X.Z.); 18481902483@163.com (Y.D.); yangliangagu@sina.com (L.Y.); 15999154824@163.com (W.R.); yugao12123@163.com (Y.G.); kangyuyao930@163.com (K.Y.); zyxgy1123@163.com (Y.Z.)

**Keywords:** valine, mTOR signaling pathway, MAC-T cells, α-casein

## Abstract

This study utilized MAC-T cells cultured in vitro as a model to investigate the effects of varying concentrations of valine on α-casein synthesis and its underlying regulatory mechanisms. In this experiment, MAC-T cells were subjected to a 12 h starvation period, followed by the addition of valine in a range of concentrations (a total of seven concentrations: 0.000, 1.596, 3.192, 6.384, 12.768, 25.536, and 51.072 mM, as well as in 10% Fetal Bovine Serum). The suitable range of valine concentrations was determined using enzyme-linked immunosorbent assays (ELISAs). Real-time fluorescent quantitative PCR (RT-qPCR) and Western blot analyses were employed to evaluate the expression levels and phosphorylation states of the casein alpha s1 gene (*CSN1S1*), casein alpha s2 gene (*CSN1S2*) and mTOR signaling pathway-related genes. The functionality of the mTOR signaling pathway was further validated through rapamycin (100.000 nM) inhibition experiments. Results indicated that 1× Val (6.384 mM), 2× Val (12.768 mM), 4× Val (25.536 mM), and 8× Val (51.072 mM) significantly enhanced α-casein synthesis (*p* < 0.01). Within this concentration range, valine significantly upregulated the expression of *CSN1S1*, *CSN1S2*, and mTOR signaling pathway-related genes including the RagA gene (*RRAGA*), RagB gene (*RRAGB*), RagC gene (*RRAGC*), RagD gene (*RRAGD*), *mTOR*, *raptor gene* (*RPTOR*), and *4EBP1 gene* (*EIF4EBP1*), *eukaryotic initiation factor 4E* (*EIF4E*), and S6 Kinase 1 (*S6K1*) (*p* < 0.01). Notably, the expression of the eukaryotic elongation factor 2 (*EEF2*) gene peaked at 1× Val (6.384 mM), while the expression of other genes reached their maximum at 4× Val (25.536 mM). Additionally, valine significantly increased the phosphorylation levels of mTOR, S6K1, 4E-binding protein-1 (4EBP1), ribosomal *protein S6* (RPS6), and eEF2 (*p* < 0.01), with the highest phosphorylation levels of mTOR, S6K1, and RPS6 observed at 4× Val (25.536 mM). Rapamycin treatment significantly inhibited mTOR phosphorylation and α-casein synthesis (*p* < 0.01); however, the addition of 4× Val (25.536 mM) partially mitigated this inhibitory effect. In conclusion, valine promotes α-casein synthesis by activating the mTOR signaling pathway, with an optimal concentration of 4× Val (25.536 mM).

## 1. Introduction

Valine, as a typical α-amino acid, exhibits distinct molecular structural features with a molecular formula of C_5_H_11_NO_2_ and a molecular weight of 117.150 g/mol. Its chemical name is 2-amino-3-methylbutanoic acid, and its chemical composition includes both an α-amino group and an α-carboxyl group, along with a unique isopropyl side chain. These structural characteristics endow valine with unique functions within biological organisms. Valine is not only an essential amino acid for livestock and poultry but also a critical precursor for casein synthesis. As animals cannot synthesize valine de novo, it must be obtained through dietary intake [1]. Both the deficiency and excess of valine can adversely affect casein synthesis, a critical consideration in dairy farming. However, it is important to note that current practices in formulating dairy cow rations do not specifically account for valine requirements. This is primarily because dairy cows largely depend on rumen microbial protein synthesis to meet their serum valine needs. However, it is important to note that current practices in formulating dairy cow rations do not specifically account for valine requirements. This is primarily because dairy cows largely depend on rumen microbial protein synthesis to meet their serum valine needs [2]. Nonetheless, as the understanding of dairy cow nutritional requirements deepens, the critical role of valine in milk protein synthesis has garnered increasing attention. This study investigates the effects of varying valine concentrations on casein synthesis in MAC-T cells through in vitro culture, providing a theoretical foundation for optimizing dairy cow diets. Previous studies have confirmed that a reduction in dietary valine decreases milk protein yield and alters its composition, indicating that valine is a critical component influencing milk protein synthesis in dairy cows [3]. Wang et al. [4] demonstrated that the addition of valine promotes β-casein synthesis in bovine mammary epithelial cells (BMECs), with this effect being dose-dependent. At the molecular mechanism level, amino acids not only serve as substrates for protein synthesis but also regulate protein synthesis by stimulating the mTORC1 complex within the mTOR signaling pathway, thereby influencing the expression of downstream genes and protein phosphorylation [5,6]. Among the amino acids, branched-chain amino acids (particularly valine) exhibit the most pronounced regulatory effects due to their unique side chain structural characteristics [7]. It is noteworthy that although α-casein and β-casein belong to the same casein family, they demonstrate significant structural and functional differentiation: α-casein forms calcium-sensitive micelle structures via N-terminal phosphorylated serine clusters, whereas β-casein performs mineral transport functions through its hydrophobic C-terminal domain [8]. These differences may result in distinct response mechanisms of the two caseins to valine. For example, the linear dose–response effect of β-casein has been well established [4], while the regulation of α-casein synthesis by the mTOR pathway phosphorylation cascade remains unclear. Therefore, investigating the effects of valine on α-casein synthesis and elucidating the underlying mechanisms is of significant theoretical and practical importance.

In this study, we employed in vitro cultured immortalized MAC-T cells as an experimental model to investigate the effects of valine on α-casein synthesis and its regulatory mechanisms. Specifically, the study focused on the following aspects: (1) the impact of different valine concentrations on α-casein synthesis and gene expression in MAC-T cells; (2) the expression of key factor genes and protein phosphorylation in the mTOR signaling pathway; and (3) the effects of adding valine after inhibiting mTOR phosphorylation on α-casein synthesis, mTOR signaling pathway-related gene expression, and protein phosphorylation levels in MAC-T cells. It is important to note that this study is based on in vitro experimental data, aiming to elucidate the mechanisms by which valine influences α-casein synthesis in MAC-T cells. This research provides a theoretical foundation for the further in vivo validation of these findings in dairy cows. While in vitro results cannot be directly extrapolated to in vivo conditions, they offer valuable insights into the function of valine in BMECs.

## 2. Results

### 2.1. Effects of Varying Valine Concentrations on α-Casein Synthesis in MAC-T Cells

**Figure 1:**
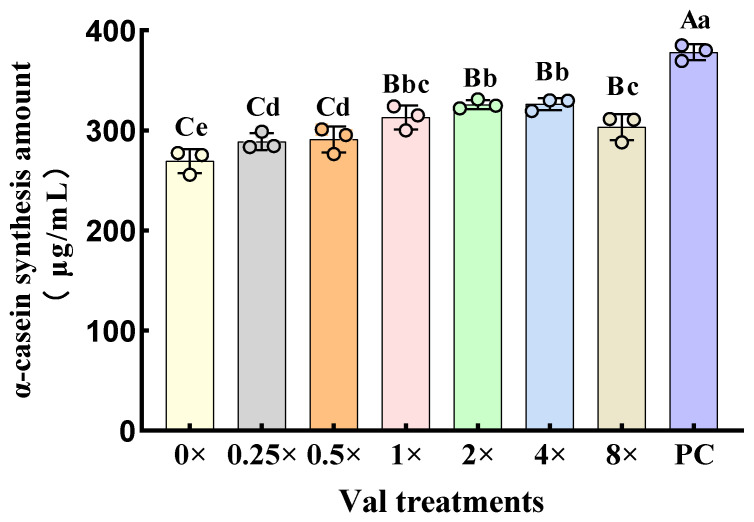
Effects of varying valine concentrations on α-casein synthesis in MAC-T Cells. The error bars represent the SD (*n* = 3). ANOVA was used to detect the differences between groups, and the LSD method was combined for uncorrected exploratory pairwise comparisons, while the Duncan method was used for conservative multiple comparison corrections. Different lowercase letters indicate significant differences (*p* < 0.05), while different uppercase letters indicate significant differences (*p* < 0.01).

Following the lysis of MAC-T cells, intracellular α-casein synthesis levels were quantified (Figure 1). The results demonstrated that, compared with the 0× Val group, the addition of valine at concentrations of 1× Val, 2× Val, 4× Val, and 8× Val significantly increased intracellular α-casein synthesis (*p* < 0.01). In the 1× Val and 0.5× Val treatment groups, valine also significantly increased α-casein synthesis (*p* < 0.05). Compared with the PC group, the amount of intracellular α-casein synthesis in MAC-T cells in the 0× Val, 0.25× Val, 0.5× Val, 1× Val, 2× Val, 4× Val, and 8× Val treatment groups was significantly reduced (*p* < 0.01). The above results demonstrate that the 1× Val, 2× Val, 4× Val, and 8× Val treatment groups significantly promoted α-casein synthesis in MAC-T cells. Therefore, these valine concentrations were selected for subsequent experiments.

### 2.2. Effects of Varying Valine Concentrations on the Relative Expression Level of CSN1S1 and CSN1S2

**Figure 2:**
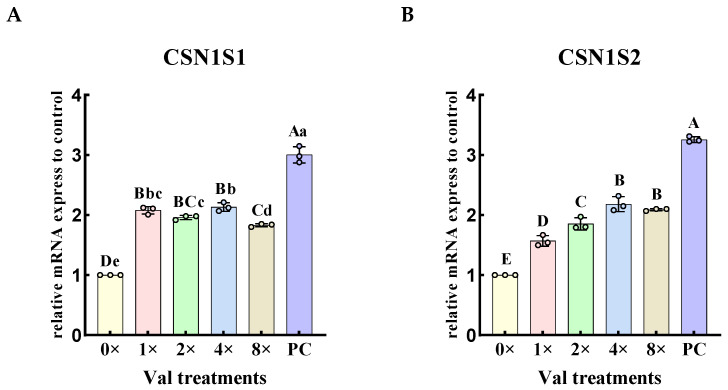
Effects of varying valine concentrations on the relative expression levels of *CSN1S1* and *CSN1S2* genes. (**A**,**B**) represent the relative mRNA expression levels of *CSN1S1* and *CSN1S2*, respectively. The error bars represent the SD (*n* = 3). ANOVA was used to detect the differences between groups, and the LSD method was combined for uncorrected exploratory pairwise comparisons, while the Duncan method was used for conservative multiple comparison corrections. Different lowercase letters indicate significant differences (*p* < 0.05), while different uppercase letters indicate significant differences (*p* < 0.01).

The relative expression levels of *CSN1S1* and *CSN1S2* genes were quantified in the four groups exhibiting the highest α-casein synthesis in MAC-T cells (Figure 2). The results demonstrated that, compared with the 0× Val group, the relative expression levels of the casein alpha s1 gene (*CSN1S1*) and casein alpha s2 gene (*CSN1S2*) were significantly increased in the 1× Val, 2× Val, 4× Val, and 8× Val treatment groups (*p* < 0.01). In the 1× Val and 4× Val treatment groups, the relative expression levels of the *CSN1S1* gene were higher than those in the other valine treatment groups and the 0× Val group (Figure 2A). Notably, the relative expression level of the *CSN1S2* gene exhibited an initial increase followed by a decrease as the valine concentration increased (Figure 2B). In the 4× Val and 8× Val treatment groups, the relative expression levels of the *CSN1S2* gene were significantly higher than those in the other valine treatment groups and the 0× Val group (*p* < 0.01). Although no significant difference was observed in *CSN1S1* gene expression between the 4× Val and 8× Val groups (*p* > 0.05), the *CSN1S2* gene expression in the 4× Val group was 4.5% higher than that in the 8× Val group. Compared with the PC group, the relative expression levels of the *CSN1S1* and *CSN1S2* genes in the 0× Val, 1× Val, 2× Val, 4× Val, and 8× Val treatment groups were significantly reduced (*p* < 0.01).

### 2.3. Effects of Varying Valine Concentrations on the Expression Levels of mTOR Signaling Pathway-Related Genes and Protein Phosphorylation

#### 2.3.1. Effects of Varying Valine Concentrations on the Expression Levels of Rag Small G Protein Genes Upstream in the mTOR Signaling Pathway

**Figure 3:**
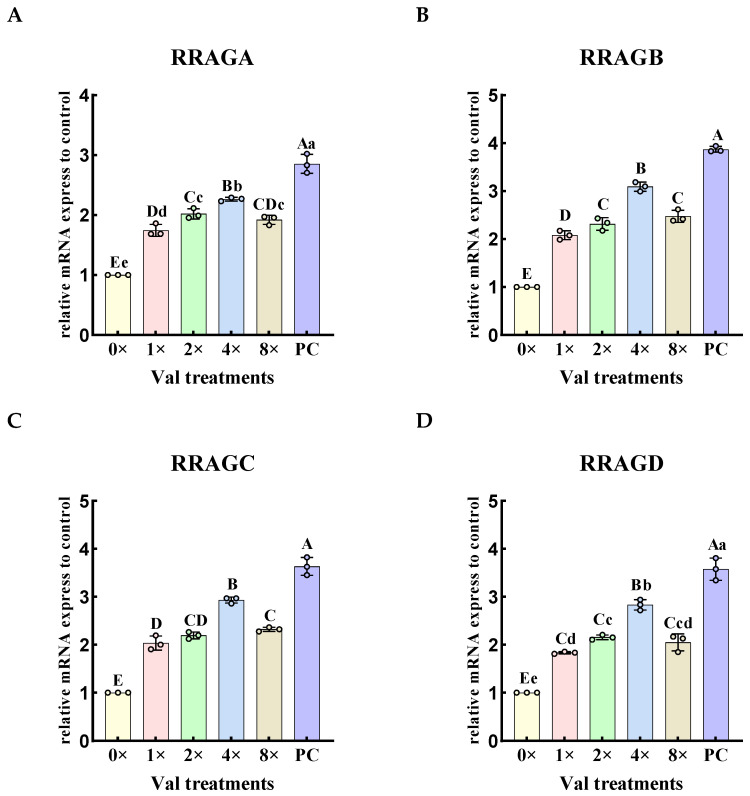
Effects of varying valine concentrations on the expression levels of Rag small G Protein genes upstream in the mTOR signaling pathway. (**A**–**D**) represent the relative mRNA expression levels of four protein genes related to Rag small G proteins upstream of the mTOR signaling pathway: *RRAGA*, *RRAGB*, *RRAGC* and *RRAGD*. The error bars represent the SD (*n* = 3). ANOVA was used to detect the differences between groups, and the LSD method was also used for uncorrected exploratory pairwise comparisons, while the Duncan method was used for conservative multiple comparison corrections. Different lowercase letters indicate significant differences (*p* < 0.05), while different uppercase letters indicate significant differences (*p* < 0.01).

The relative expression levels of Rag small G protein genes upstream in the mTOR signaling pathway were quantified in the four groups exhibiting the highest α-casein synthesis in MAC-T cells (Figure 3). The results demonstrated that, valine in all experimental groups significantly increased the relative expression of the RagA gene (*RRAGA*), RagB gene (*RRAGB*), RagC gene (*RRAGC*), and RagD gene (*RRAGD*) (*p* < 0.01), and showed a tendency of increasing and then decreasing, where the relative expression of all genes was the highest in the 4× Val treatment group. Compared with the PC group, the relative expression levels of *RRAGA*, *RRAGB*, *RRAGC*, and *RRAGD* genes in the 0× Val, 0.25× Val, 0.5× Val, 1× Val, 2× Val, 4× Val, and 8× Val treatment groups were significantly reduced (*p* < 0.01). The “highest expression level” only refers to the relatively optimal value in the valine concentration gradient experiment, rather than exceeding that of the FBS group.

#### 2.3.2. Effects of Varying Valine Concentrations on the Relative Expression Levels of mTORC1-Related Genes

**Figure 4:**
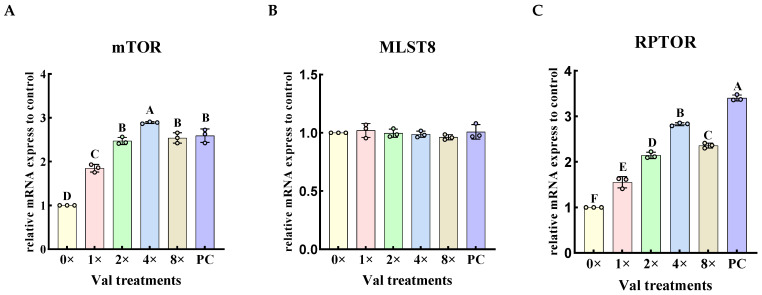
Effects of varying valine concentrations on the relative expression levels of mTORC1 complex-related genes. The error bars represent the SD (*n* = 3). (**A**–**C**) represent the relative mRNA expression levels of the mTORC1 complex-related protein genes, *mTOR*, *MLST8*, and *RPTOR*, respectively. ANOVA was used to detect the differences between groups, and the LSD method was combined for uncorrected exploratory pairwise comparisons, while the Duncan method was used for conservative multiple comparison corrections. Different uppercase letters indicate significant differences (*p* < 0.01).

The relative expression levels of mTORC1-related genes upstream in the mTOR signaling pathway were quantified in the four groups exhibiting the highest α-casein synthesis in MAC-T cells (Figure 4). The results demonstrated that, compared with the 0× Val group, the addition of valine at concentrations of 1× Val, 2× Val, 4× Val, and 8× Val significantly increased the relative expression levels of the *mTOR* and raptor gene (*RPTOR*) (Figure 4A,C, *p* < 0.01). The expression levels of both *mTOR* and *RPTOR* genes initially increased and then decreased with increasing valine concentrations, reaching their highest levels in the 4× Val treatment group. In contrast, the addition of valine at these concentrations had no significant effect on the expression level of the *GβL gene* (*MLST8*) (Figure 4B, *p* > 0.05). Compared with the PC group, the relative expression of the *mTOR* gene in the 4× Val treatment group was significantly upregulated (*p* < 0.01), with an 11.5% increase. The relative expression of the *RPTOR* gene was significantly down-regulated in the 0× Val, 1× Val, 2× Val, 4× Val, and 8× Val treatment groups (*p* < 0.01). The “highest expression level of *RPTOR*” observed in the valine concentration gradient experiment refers to its relatively optimal value within this specific experimental context, and does not exceed that of the FBS group.

#### 2.3.3. Effects of Varying Valine Concentrations on the Relative Expression Levels of Downstream Genes in the mTOR Signaling Pathway

**Figure 5:**
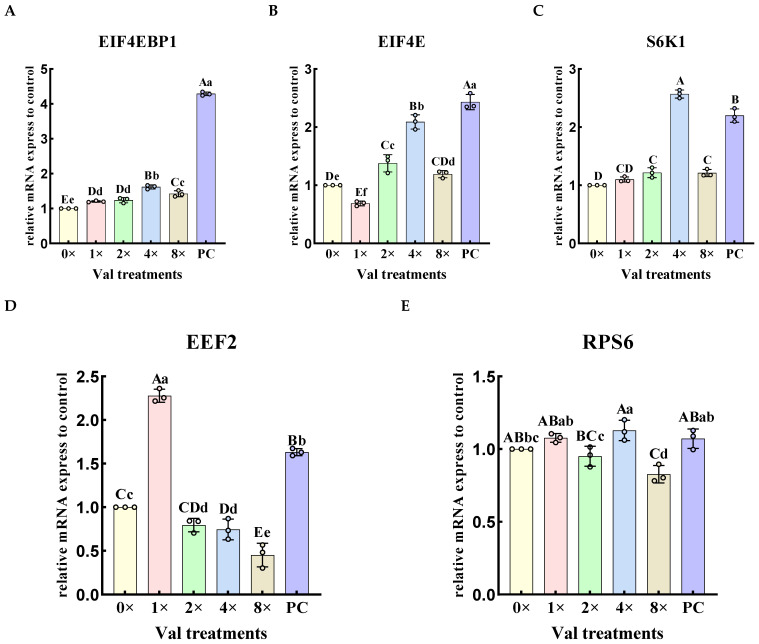
Effects of varying valine concentrations on the relative expression levels of downstream genes in the mTOR signaling pathway. (**A**–**E**) represent the relative mRNA expression levels of the five key downstream targets of the mTOR pathway: *EIF4EBP1*, *EIF4E*, *S6K1*, *EEF2*, and *RPTOR*. The error bars represent the SD (*n* = 3). ANOVA was used to detect the differences between groups, and the LSD method was combined for uncorrected exploratory pairwise comparisons, while the Duncan method was used for conservative multiple comparison corrections. Different lowercase letters indicate significant differences (*p* < 0.05), while different uppercase letters indicate significant differences (*p* < 0.01).

The relative expression levels of downstream genes in the mTOR signaling pathway were quantified in the four groups exhibiting the highest α-casein synthesis in MAC-T cells (Figure 5). The results demonstrated that, compared with the 0× Val group, the addition of valine at concentrations of 1× Val, 2× Val, 4× Val, and 8× Val significantly upregulated the expression levels of the 4EBP1 gene (*EIF4EBP1*) (Figure 5A, *p* < 0.01), with the 4× Val group exhibiting the most pronounced effect on the relative expression level of the *EIF4EBP1* gene. The expression levels of the *eukaryotic initiation factor 4E* (*EIF4E*) and S6 Kinase (*S6K1*) genes initially increased and then decreased as valine concentrations increased, reaching their highest levels in the 4× Val group, which were significantly higher than those in the 0× Val group and other valine concentration groups (Figure 5B,C, *p* < 0.01). The expression level of the eukaryotic elongation factor 2 (*EEF2*) gene progressively decreased with increasing valine concentrations, being significantly higher in the 1× Val group compared to all other groups (Figure 5D, *p* < 0.01). In contrast, the expression level of the ribosomal protein S6(*RPS6*) gene was significantly higher in the 4× Val treatment group, where valine was added, compared to both the 2× Val and 8× Val treatment groups (Figure 5E, *p* < 0.01), and it was significantly higher than that in the 0× Val group (*p* < 0.05). When valine was added in the 8× Val treatment group, the expression level of the *RPS6* gene was significantly lower than that in the 0× Val, 1× Val and 4× Val treatment groups (*p* < 0.01), and it was significantly lower than that in the 2× Val treatment group (*p* < 0.05). Compared with the PC group, the relative expression levels of *EIF4EBP1* and *EIF4E* genes were significantly down-regulated in all treatment groups (0× Val, 1× Val, 2× Val, 4× Val, and 8× Val; *p* < 0.01). The relative expression of the *S6K1* gene was significantly up-regulated in the 4× Val treatment group (*p* < 0.01), with a 16.8% increase compared to the PC group. The relative expression of the *EEF2* gene was significantly up-regulated in the 1× Val treatment group (*p* < 0.01), with a 39.6% increase compared to the PC group. Additionally, the relative expression of the *RPS6* gene was significantly down-regulated in the 2× Val experimental group (*p* < 0.05) and significantly down-regulated in the 8× Val treatment group (*p* < 0.01). The “highest expression levels of *EIF4EBP1* and *EIF4E*” observed in the valine concentration gradient experiment refer to their relative optimal values within this specific experimental context, and do not exceed those of the FBS group.

#### 2.3.4. Effects of Varying Valine Concentrations on the Phosphorylation of Downstream Proteins in the mTOR Signaling Pathway

**Figure 6:**
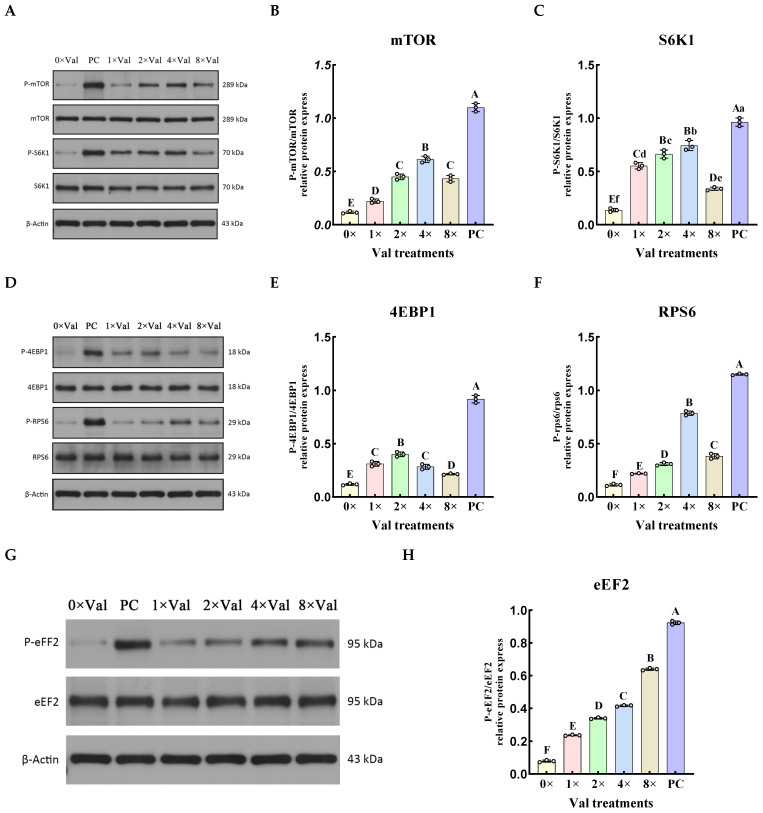
Effects of different valine concentrations on the phosphorylation of downstream proteins in the mTOR signaling pathway. (**A**) Western blotting was used to detect the protein expression levels of phosphorylated mTOR (P-mTOR) and phosphorylated S6K1 (P-S6K1). (**B**) The phosphorylation level of mTOR protein (P-mTOR/mTOR) was quantified and calculated using β-actin and mTOR as internal references. (**C**) The phosphorylation level of S6K1 protein (P-S6K1/S6K1) was quantified and calculated using β-actin and S6K1 as internal references. (**D**) Western blotting was used to detect the protein expression levels of phosphorylated 4EBP1 (P-4EBP1) and phosphorylated RPS6 (P-RPS6). (**E**) The phosphorylation level of 4EBP1 protein (P-4EBP1/4EBP1) was quantified and calculated using β-actin and 4EBP1 as internal references. (**F**) The phosphorylation level of RPS6 protein (P-RPS6/RPS6) was quantified and calculated using β-actin and RPS6 as internal references. (**G**) Western blotting was used to detect the protein expression levels of phosphorylated eEF2 (P-eEF2). (**H**) The phosphorylation level of eEF2 protein (P-eEF2/eEF2) was quantified and calculated using β-actin and eEF2 as internal references. The error bars represent the SD (*n* = 3). ANOVA was used to detect the differences between groups, and the LSD method was combined for uncorrected exploratory pairwise comparisons, while the Duncan method was used for conservative multiple comparison corrections. Different lowercase letters indicate significant differences (*p* < 0.05), while different uppercase letters indicate significant differences (*p* < 0.01).

The above results suggest that supplementation with optimal levels of valine enhances the expression of genes associated with mTOR signaling pathway proteins. To further substantiate that valine activates the mTOR signaling pathway, we examined the phosphorylation levels of key proteins in this pathway across six groups: 0× Val, 1× Val, 2× Val, 4× Val, 8× Val, and PC (Figure 6). The results revealed that the phosphorylation levels of mTOR, S6K1, 4E-binding protein-1 (4EBP1), and RPS6 proteins initially increased and then decreased with increasing valine concentrations (Figure 6B,C,E,F). Specifically, in the 4× Val valine group, the phosphorylation levels of mTOR, S6K1, and RPS6 proteins reached their peak. Notably, the phosphorylation levels of mTOR and RPS6 were significantly higher than those in the 0× Val group and other valine groups (*p* < 0.01), while the phosphorylation level of RPS6 was also significantly higher than that in the 0× Val group and other valine groups (*p* < 0.05). In the 2× Val valine group, the phosphorylation level of 4EBP1 protein was significantly higher compared to that in the 0× Val group and other valine groups (*p* < 0.01). Conversely, the phosphorylation level of eEF2 protein progressively increased with increasing valine concentrations, reaching a significant peak in the 8× Val valine group, which was markedly higher than that in the 0× Val group and other valine groups (Figure 6H, *p* < 0.01). Compared with the PC group, the phosphorylation levels of mTOR, S6K1, 4EBP1, RPS6, and eEF2 were significantly reduced in the 0× Val, 1× Val, 2× Val, 4× Val, and 8× Val treatment groups. The “highest expression level” only refers to the relatively optimal value in the valine concentration gradient experiment, rather than exceeding that of the FBS group.

### 2.4. To Investigate the Role of the mTOR Signaling Pathway in Valine-Induced α-Casein Synthesis in MAC-T Cells by Inhibiting mTOR

**Figure 7:**
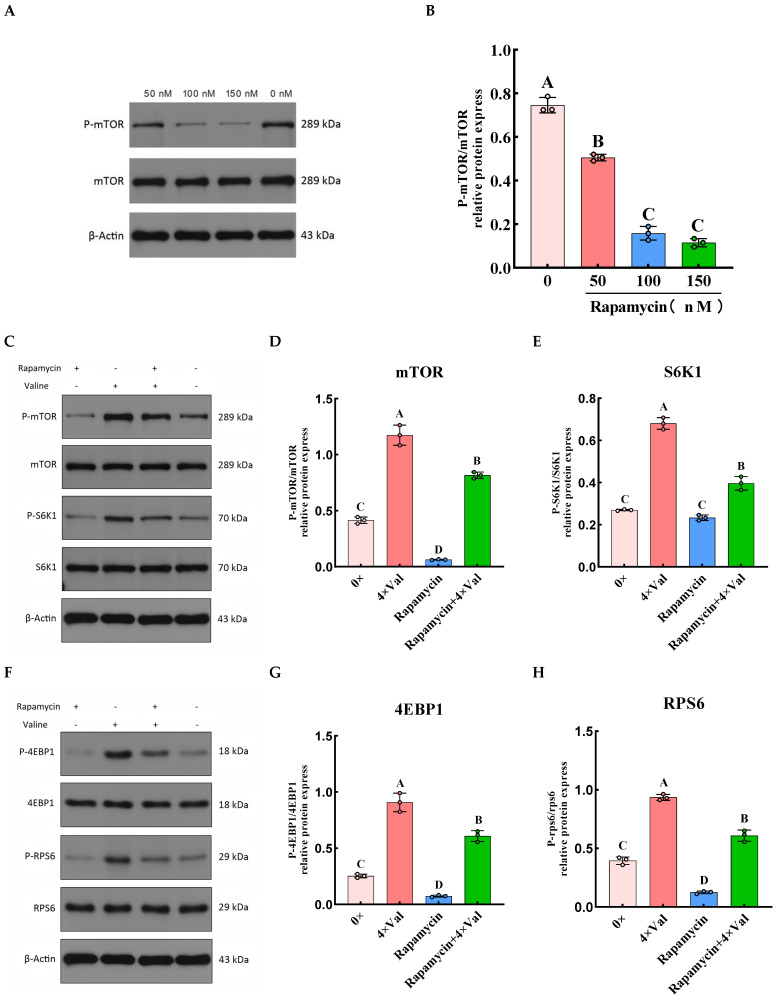

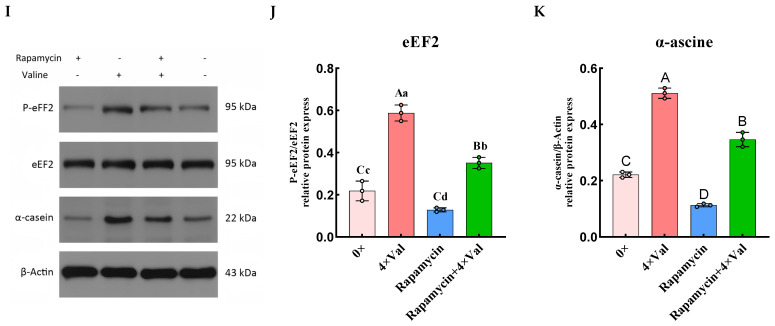
Effects of valine and rapamycin on the phosphorylation levels of mTOR downstream proteins. (**A**) The phosphorylation levels of mTOR (P-mTOR) in MAC-T cells treated with varying concentrations of rapamycin were assessed using Western blotting. (**B**) The P-mTOR level (P-mTOR/mTOR) in MAC-T cells treated with 100 nM rapamycin was quantified and calculated using β-actin and mTOR as internal references. (**C**) Western blotting was used to detect the protein expression levels of P-mTOR and P-S6K1. (**D**) The P-mTOR level (P-mTOR/mTOR) was quantified and calculated using β-actin and mTOR as internal references. (**E**) The P-S6K1 level (P-S6K1/S6K1) was quantified and calculated using β-actin and S6K1 as internal references. (**F**) Western blotting was used to detect the protein expression levels of P-4EBP1 and P-RPS6. (**G**) The P-4EBP1 level (P-4EBP1/4EBP1) was quantified and calculated using β-actin and 4EBP1 as internal references. (**H**) The P-RPS6 level (P-RPS6/RPS6) was quantified and calculated using β-actin and RPS6 as internal references. (**I**) Western blotting was used to detect the protein expression levels of P-eEF2 and α-casein. (**J**) The P-eEF2 level (P-eEF2/eEF2) was quantified and calculated using β-actin and eEF2 as internal references. (**K**) The α-casein protein expression level was quantified using β-actin as an internal reference. The error bars represent the SD (*n* = 3). ANOVA was used to detect the differences between groups, and the LSD method was combined for uncorrected exploratory pairwise comparisons, while the Duncan method was used for conservative multiple comparison corrections. Different lowercase letters indicate significant differences (*p* < 0.05), while different uppercase letters indicate significant differences (*p* < 0.01).

The above results suggest that valine may promote α-casein synthesis by activating the mTOR signaling pathway. To verify this hypothesis, we first determined the optimal inhibitory concentration of rapamycin. Subsequently, we added rapamycin to the selected experimental groups and measured the phosphorylation levels of key downstream proteins in the mTOR signaling pathway (Figure 7). The results revealed that compared with the 0× Val, adding 50, 100, and 150 nM of rapamycin to MAC-T cells significantly inhibited mTOR phosphorylation (Figure 7B, *p* < 0.01). Among these concentrations, 100 nM and 150 nM rapamycin exhibited the strongest inhibitory effects on mTOR phosphorylation, but there was no significant difference between them (*p* > 0.05). Due to the cytotoxic effects of rapamycin, 100 nM rapamycin was selected for subsequent experiments. Compared with the 0× Val group, 100 nM rapamycin significantly reduced the phosphorylation levels of mTOR, 4EBP1, and RPS6, as well as α-casein synthesis (Figure 7D,G,H,K, *p* < 0.01); it also significantly decreased eEF2 phosphorylation (Figure 7J, *p* < 0.05), while having no significant effect on S6K1 phosphorylation (Figure 7E, *p* > 0.05). However, the addition of valine to the culture medium containing rapamycin mitigated its inhibitory effects. Specifically, compared with the rapamycin group, valine addition significantly increased the phosphorylation levels of mTOR, S6K1, 4EBP1, RPS6, and eEF2, as well as α-casein synthesis (*p* < 0.01).

## 3. Discussion

### 3.1. Effects of Different Concentrations of Valine on the Synthesis of α-Casein

Research has demonstrated that over 90% of de novo protein synthesis in the mammary gland utilizes free amino acids from serum as substrates [9]. Amino acids serve not only as precursors for milk protein synthesis but also as regulatory factors [10]. The concentration of amino acids directly influences the rate of milk protein synthesis [11]. When amino acid levels are low, MAC-T cells reduce the rate of milk protein synthesis to adapt to limited amino acid availability. Conversely, when amino acid levels are high, these cells increase the rate of milk protein synthesis to maximize amino acid utilization. In this study, we found that adding of 1× Val, 2× Val, 4× Val, and 8× Val significantly enhanced intracellular α-casein synthesis, with a trend of initially increasing and then decreasing as the valine concentration increased. Notably, there was no significant difference in the synthesis of intracellular α-casein between the experimental groups supplemented with 0.25× Val and 0.5× Val, while the group supplemented with 4× Val exhibited the highest synthesis of intracellular α-casein. This suggests that at lower valine concentrations (0.25× Val and 0.5× Val), MAC-T cells were still in an adaptation phase to exogenous nutrients, resulting in relatively low α-casein synthesis rates. In contrast, when 4× Val was added, the MAC-T cells maximized the utilization of valine, resulting in the highest synthesis rate of α-casein. This further underscores the pivotal role of amino acids as both precursors and regulatory factors in milk protein synthesis. In practical livestock applications, this finding has significant implications for optimizing feed formulations for both ruminants and monogastric animals. For ruminants, which heavily depend on microbial protein to meet their amino acid requirements [12], precise regulation of dietary valine levels could potentially enhance milk yield and quality. For monogastric animals, where diet directly influences serum amino acid levels, this study provides a theoretical foundation for using nutritional interventions to regulate serum amino acid concentrations, thereby optimizing milk protein synthesis. Additionally, this study observed a trend where α-casein synthesis initially increases and then decreases as valine levels rise. Specifically, when valine concentrations exceed optimal levels, α-casein synthesis decreases. This phenomenon may be attributed to the dose-dependent nature of valine’s promoting effect on α-casein synthesis, where excessive valine supply reduces its utilization efficiency. Wang et al. [13] demonstrated that the optimal concentration range for arginine is 369.65 to 434.44 mg/L. Exceeding this range leads to a decline in casein synthesis efficiency, which aligns with the trends observed in our study. The above results indicate that, in practical applications, precise control of amino acid supplementation is essential to optimize production efficiency.

### 3.2. Effects of Varying Valine Concentrations on the Relative Expression Level of the α-Casein Gene

Amino acids not only serve as precursor substances for the synthesis of milk proteins but also function as signaling molecules. They regulate the transcription and translation of milk protein genes through multiple signaling pathways in mammary epithelial cells, thereby promoting casein synthesis [14]. A deficiency in branched-chain amino acids may down-regulate the expression of casein-coding genes and inhibit milk protein synthesis [15]. According to Hao [16], adding 0.900 mM valine to BMECs significantly upregulated the relative expression levels of casein-encoding genes (*CSN1S1*, *CSN2*, and *CSN3*). In contrast, both low concentrations (0.270 and 0.360 mM) and high concentrations (1.800 mM) of valine had no significant effect on the relative expression levels of these genes. The results of this study demonstrate that the addition of 1× Val, 2× Val, 4× Val, and 8× Val all significantly upregulate the relative expression levels of the *CSN1S1* and *CSN1S2* genes. However, the effect of different levels of valine on the upregulation of these genes’ relative expression varies. The relative expression level of the *CSN1S1* gene was higher in both the 1× Val and 4× Val treatment groups compared to the other two valine-supplemented groups and the 0× Val group. Similarly, the relative expression level of the *CSN1S2* gene was higher in the 4× Val and 8× Val treatment groups compared to the other two valine-supplemented groups and the 0× Val group. The above results indicate that valine can promote the relative expression of α-casein-encoding genes, and there is an optimal level of valine that promotes the expression of these related genes. Specifically, the 4× Val treatment group demonstrates the best promotional effect of valine on α-casein synthesis in MAC-T cells.

### 3.3. Effects of Varying Valine Concentrations on the Expression and Phosphorylation Levels of Genes Related to α-Casein Synthesis Mediated by the mTOR Signaling Pathway

Studies have shown that the mechanism by which valine stimulates casein synthesis in BMECs may be associated with the activation of the mTOR signaling pathway [17]. mTOR is a critical regulator of milk protein synthesis in mammary tissue and primarily involves two complexes: mTORC1 and mTORC2 [18]. Specifically, mTORC1 promotes protein synthesis by facilitating mRNA translation [19]. According to Appuhamy [20], essential amino acids influence milk protein synthesis in mammary epithelial cells by modulating the mTOR signaling pathway. Upon entering the cells, amino acids act as signaling molecules, upregulating the expression of upstream genes related to mTOR and its signaling pathway [21]. They also activate the Rag small G proteins by altering their guanine nucleotide binding state. The activated Rag small G proteins specifically bind to RPTOR in the mTORC1 complex, translocating it to the lysosome and activating mTORC1, which subsequently phosphorylates proteins involved in the mTOR signaling pathway, thereby promoting casein synthesis [22]. In this study, we found that adding 1× Val, 2× Val, 4× Val and 8× Val upregulated the expression of Rag small G protein-related genes (*RRAGA*, *RRAGB*, *RRAGC*, *RRAGD*) as well as mTORC1 complex-related genes (*mTOR* and *RPTOR*) upstream of the mTOR signaling pathway, while also increasing the phosphorylation level of mTOR. This suggests that the Rag small G proteins were in an activated state under these conditions, and the addition of valine stimulated the activation of mTORC1 via the Rag small G proteins. Notably, as a component of the mTORC1 complex, the gene expression level of *mTOR* was higher in the 4× Val treatment group compared to the 10% FBS group, while the expression level of the *MLST8* gene remained insignificant across all groups with varying levels of valine addition. Possibly because mTOR acts as a crucial factor in amino acid sensing and signal transduction, the expression of the *mTOR* gene is more sensitive to the stimulation by valine. When 4× Val is added, the intracellular nutrients become more abundant, resulting in the high expression of the *mTOR* gene. In contrast, *GβL*, as a component of the mTORC1 complex, exhibited relatively stable expression levels and was less responsive to changes in valine concentration.

Studies have shown that when mTOR is activated, it phosphorylates two parallel downstream proteins with mRNA translation regulatory functions: S6K1 and 4EBP1. This activation leads to the phosphorylation of a series of downstream regulatory proteins, including eIF4E, RPS6, and eEF2, thereby promoting the initiation of mRNA translation [23,24,25]. Unphosphorylated 4EBP1 binds to eIF4E, inhibiting protein synthesis by suppressing translation initiation. mTORC1 relieves this inhibition by phosphorylating 4EBP1, reducing its binding affinity for eIF4E [26]. Increased phosphorylation of S6K1 promotes the phosphorylation of its downstream targets, RPS6 and eEF2. Notably, RPS6 phosphorylation directly reflects the activation state of the mTORC1 pathway [27], while eEF2 phosphorylation can impede peptide chain elongation, thereby decreasing the rate of protein synthesis [28,29]. This study revealed that the expression levels and phosphorylation status of both *EIF4EBP1* and *S6K1* genes were significantly increased when 1× Val, 2× Val, 4× Val, and 8× Val were added. Moreover, in the 4× Val treatment group, the expression level of the *S6K1* gene was higher than that in the 10% FBS group. This is possibly due to the fact that both the expression level of the *mTOR* gene and the protein phosphorylation level are highest in the 4× Val treatment group, and that the mediation of 4EBP1 phosphorylation by essential amino acids is independent of mTOR [30], which leads to the expression level of the *S6K1* gene, a downstream target of mTOR, being higher in the 4× Val treatment group compared to the 10%FBS group. Furthermore, the phosphorylation level of S6K1 is higher than that of 4EBP1 in the 1× Val, 2× Val, 4× Val, and 8× Val treatment groups. Our findings also indicate that valine addition differentially affects the expression and protein phosphorylation levels of key factors downstream of 4EBP1 and S6K1, namely *EIF4E*, *EEF2*, and *RPS6* genes. The expression level of the *EIF4E* gene reached its lowest point in the 1× Val treatment group and was lower than that in the 0× Val group. In contrast, the expression level of the *EEF2* gene gradually increased with increasing valine concentrations, and in the 1× Val treatment group, the expression level of the *EEF2* gene was higher than that in the 0× Val group. However, the phosphorylation level of eEF2 protein progressively decreased as valine concentration increased. The expression level of the *RPS6* gene was only higher in the 4× Val treatment group compared to the 0× group, whereas the phosphorylation level of the RPS6 protein was significantly increased in the 1× Val, 2× Val, 4× Val, and 8× Val treatment groups, with the highest phosphorylation level observed in the 4× Val treatment group. These results suggest that when 1× Val was added, MAC-T cells were in an intermediate state of response to valine. Activation of the mTOR signaling pathway increased the expression and phosphorylation levels of the *EIF4EBP1* gene, but other regulatory mechanisms limited the gene expression of *EIF4E*, leading to its lower expression compared to that of the 0× Val group. At this concentration, MAC-T cells upregulated the expression of the *EEF2* gene to maximize valine utilization. As valine levels increased, MAC-T cells accumulated sufficient valine for α-casein synthesis, subsequently down-regulating *EEF2* gene expression and increasing eEF2 phosphorylation to reduce α-casein synthesis rates. The addition of valine had a weak effect on *RPS6* gene expression levels, whereas the effect on RPS6 phosphorylation levels was more significant, indicating that in the regulation of protein function, post-translational modifications (such as phosphorylation) often have a more direct and critical influence on protein function and activity than changes in gene expression levels. The phosphorylation level of RPS6 was highest in the 4× Val treatment group, likely due to the valine-induced activation of the mTOR signaling pathway. This activation subsequently enhanced the phosphorylation of the downstream target S6K1, which in turn further promoted RPS6 phosphorylation. Consequently, this cascade led to increased ribosomal activity and facilitated α-casein synthesis.

Meanwhile, this study also found that the addition of 1× Val, 2× Val, 4× Val, and 8× Val all upregulated the relative expression levels of Rag small G protein genes (*RRAGA*, *RRAGB*, *RRAGC*, *RRAGD*) upstream of mTOR and mTORC1 complex-related genes (*mTOR* and *RPTOR*), and downstream of mTOR signaling pathway-related genes (*EIF4EBP1*, *EIF4E*, *S6K1*, *RPS6*). Additionally, within this concentration range, valine also enhanced the phosphorylation levels of downstream mTOR signaling pathway-related proteins (mTOR, S6K1, 4EBP1, and RPS6). The expression levels of mTOR signaling pathway-related genes and the phosphorylation levels of related proteins initially increased and then decreased with increasing valine concentration, reaching their peak in the 4× Val treatment group. This could be due to the dose-dependent effect of valine on upregulating genes related to the mTOR signaling pathway and promoting the phosphorylation of associated proteins in MAC-T cells. When 4× Val is added, the upregulation of mTOR signaling pathway-related gene expression and the promotion of related protein phosphorylation were most effective. However, higher valine concentrations inhibited mTOR signaling pathway activation. Furthermore, treatment results showed that the upregulation of mTOR-related gene expression and the increase in related protein phosphorylation levels were most pronounced in the 10% FBS group compared to all the valine treatment groups. This phenomenon can be attributed to the significant activation of the mTOR signaling pathway by 10% FBS, likely due to its complex multi-component synergistic effects [31]. In addition to valine, FBS is rich in other essential amino acids (such as leucine and isoleucine), growth factors (such as IGF-1 and EGF), and hormones (such as insulin). These components can synergistically activate the mTOR signaling pathway through multiple mechanisms, including the PI3K/Akt pathway [32,33]. In contrast, while valine supplementation alone can partially restore the inhibited signaling under serum-free conditions, its effectiveness is limited by the absence of other synergistic factors. This study also found that even when 4× Val was supplemented in serum-free medium, it could not fully replicate the pro-coalescence effect of FBS. This suggests that in breast epithelial cells, valine is a necessary but not sufficient condition for mTOR pathway activation. Future research should further investigate the combined effects of valine with other nutrients (such as leucine and glucose) or growth factors.

### 3.4. To Investigate the Role of the mTOR Signaling Pathway in Valine-Induced α-Casein Synthesis in MAC-T Cells, We Conducted Experiments to Inhibit the mTOR Signaling Pathway

The mTOR signaling pathway is a critical regulator of casein synthesis. Valine has been shown to modulate protein and lipid metabolism through the mTOR signaling pathway [34,35]. Previous studies have demonstrated that adding valine can upregulate mTOR signaling, activate the mTOR pathway, and promote α-casein synthesis. To further investigate the role of the mTOR signaling pathway in valine-induced α-casein synthesis in MAC-T cells, this study used rapamycin to inhibit mTOR phosphorylation and examined changes in α-casein synthesis and the phosphorylation levels of downstream proteins in the mTOR signaling pathway. Our findings revealed that adding rapamycin alone to the MAC-T cell culture medium significantly inhibited the phosphorylation levels of mTOR, eEF2, RPS6, and 4EBP1, as well as α-casein synthesis. The inhibition was most pronounced for mTOR phosphorylation but less significant for S6K1 phosphorylation. This indicates that rapamycin, as an inhibitor of the mTOR signaling pathway, effectively suppresses mTOR phosphorylation [36], leading to the reduced phosphorylation of downstream proteins and decreased α-casein synthesis. These results directly demonstrate that a decline in mTOR signaling activity results in reduced α-casein synthesis, confirming the crucial role of the mTOR signaling pathway in promoting α-casein synthesis. The insignificant inhibitory effect on S6K1 phosphorylation may be attributed to the lag in S6K1 dephosphorylation, which is a relatively slow process. When mTOR is dephosphorylated, S6K1 can maintain its phosphorylated state for some time. Furthermore, when both rapamycin and 4× Val valine were added to MAC-T cells for 12 h, it partially mitigated the inhibitory effects of rapamycin on the phosphorylation of mTOR, eEF2, RPS6, 4EBP1, and S6K1, and increased α-casein synthesis. This suggests that the mTOR signaling pathway plays a vital role in valine-promoted α-casein synthesis in MAC-T cells, and that valine can counteract the inhibitory effects of rapamycin by restoring mTOR signaling activity, thereby enhancing α-casein synthesis. These findings underscore the dual role of valine in regulating milk protein synthesis: not only does it serve as a substrate for α-casein synthesis, but it also acts as a signaling molecule to activate the mTOR signaling pathway, thus participating in the synthesis process of α-casein.

## 4. Materials and Methods

### 4.1. Materials

In this study, L-Val was purchased from Sigma (Cat No. V0513, sigma, St. Louis, Mo, USA). Dulbecco’s Modified Eagle Medium (DMEM, Cat No. C11965500BT, Thermo Scientific, Waltham, MA, USA), Fetal Bovine Serum (FBS, Cat No. 10099-141C, Thermo Scientific, Waltham, MA, USA) and 0.25% Trypsin-EDTA (Cat No. 25200-056, Thermo Scientific, Waltham, MA, USA) were purchased from Thermo Scientific. Rapamycin was purchased from Selleck (Cat No. S1039, Selleck Chemicals LLC, Houston, TX, USA). PBS was purchased from Pricella (Cat No. Cat No. PB180327, Wuhan Pricella Biotechnology Co., Ltd., Wuhan, China).

### 4.2. MAC-T Cells

The MAC-T cells utilized in this study was obtained from the cell bank of Qingqi (Shanghai) Biotechnology Co., Ltd. (Shanghai, China). This cell line exhibits typical biological characteristics of BMECs, including stable adherent growth properties and a monolayer cobblestone-like mosaic arrangement with tight intercellular connections and clear boundaries after continuous passage culture in the laboratory. Over 30 passages, the cell population maintained >95% morphological uniformity without any observed abnormalities such as vacuolation or fibrosis. These verified characteristics indicate that this cell model can reliably mimic the synthetic and metabolic activities of mammary epithelial cells during lactation, fully meeting the requirements for constructing the experimental system in this study.

### 4.3. Experimental Design

Using in vitro cultured MAC-T cells as the experimental model, a single-factor randomized experimental design was employed. Third-generation MAC-T cells were initially cultured for 24 h and then subjected to 12 h of starvation treatment before being randomly divided into 12 groups, with three replicates per group. The positive control group (PC group) was cultured in medium containing 10% FBS, while the negative control group (0× Val group) was cultured in serum-free medium. Experimental groups received different concentrations of valine, rapamycin, or a combination of rapamycin and the optimal level of valine, based on the negative control group. Samples were collected after an additional 12 h of cell culture. The enzyme-linked immunosorbent assay (ELISA) was used to measure α-casein synthesis in the PC group, 0× Val group, and experimental groups with varying levels of valine, to determine the appropriate range of valine addition. Simultaneously, as described [37], real-time fluorescent quantitative PCR (RT-qPCR) was performed to detect the relative expression levels of the α-casein encoding gene and genes related to the mTOR signaling pathway. Western blot analysis was conducted as described [38] to assess the phosphorylation of downstream proteins in the mTOR signaling pathway and the synthesis of α-casein. By integrating the results from RT-qPCR and Western blot, the effects of different valine concentrations on α-casein synthesis and the mTOR signaling pathway were analyzed to determine the optimal valine concentration. Additionally, Western blot analysis was performed on the 0× Val group, rapamycin group, optimal valine level group, and the group treated with both rapamycin and the optimal valine level. Samples were collected after 12 h of cell culture to evaluate the phosphorylation levels of downstream mTOR proteins and α-casein synthesis. The experimental groupings are detailed in Table 1, with each group undergoing three replicate evaluations.

### 4.4. Detection Indicators and Methods

#### 4.4.1. Determination of the Synthesis Quantity of α-Casein

In accordance with the ELISA method for determining β-lactoglobulin (β-LG) as described by Pu, P. et al. [39], this study employed the ELISA technique to quantify the synthesis of α-casein in MAC-T cells. The detailed procedure is outlined as follows: After routine trypsinization, third-generation MAC-T cells were centrifuged at 1000 r/min for 10 min using a low-speed benchtop centrifuge (Cat No. LT53, Hunan Xiangyi Laboratory Instrument Development Co., Ltd., Changsha, Hunan, China) to collect the cell pellet. The cells were then resuspended in DMEM medium supplemented with 10% fetal bovine serum (FBS) and counted using a Countess 3 FL automated cell counter (Cat No. AMQAF2000, Thermo Scientific, Waltham, MA, USA). Cells were seeded into T25 culture flasks at a density of 6 × 10^6^ cells per flask and incubated at 37 °C with 5% CO_2_ (Cat No. WCI-180, Wiggens GmbH, Baden, Württemberg, Germany) for 24 h. Following this, the cells were subjected to starvation treatment with serum-free DMEM medium for 12 h [40]. The medium was then replaced with valine-containing DMEM at specified concentrations (0× Val, 0.25× Val, 0.5× Val, 1× Val, 2× Val, 4× Val, and 8× Val). After an additional 12 h of culture, the cells were collected and lysed using an ultrasonic cell disruptor (Cat No. FB50, Thermo Scientific, Waltham, MA, USA) operating at 80 kHz for 5 min. To measure α-casein synthesis, a Bovine α-Casein ELISA Kit (Cat No. JL22469, Shanghai Jianglai Biotechnology Co., Ltd., Shanghai, China) was used according to the manufacturer’s instructions. Standards and test samples (three replicate wells per group) were added to the microtiter plate, followed by 100 µL of horseradish peroxidase-labeled antibody in each well except the blank wells. The plate was covered and incubated at 37 °C in a water bath for 1 h in the dark. After incubation, the liquid was discarded, and each well was washed five times with 350 µL of wash buffer, standing for 3 min between washes. The wells were patted dry on absorbent paper after each wash. Next, 50 µL of Substrate A and B were added to each well, and the plate was incubated at 37 °C in a water bath for 15 min in the dark. Finally, 50 µL of stop solution was added to each well, and the OD value was measured at 450 nm within 15 min using a Multimode Microplate Reader (Cat No. Infinite 200Pro, Tecan GmbH, Männedorf, Switzerland). Intracellular α-casein synthesis was quantified based on the standard curve. Based on the ELISA results, the four amino acid level groups with the highest intracellular α-casein synthesis were selected for subsequent experiments.

#### 4.4.2. Determination of the Expression Levels of Related Genes

To extract total cellular RNA, the Trizol method was employed with the following specific procedures: After routine trypsinization of third-generation MAC-T cells, they were centrifuged at 1000 r/min for 10 min to collect the cell pellet. The cells were resuspended in DMEM medium supplemented with 10% fetal bovine serum (FBS) and counted using a Countess 3 FL automated cell counter (Cat No. AMQAF2000, Thermo Scientific, Waltham, MA, USA). The cell suspension was seeded into a 6-well plate at a density of 3 × 10^5^ cells per well and incubated at 37 °C with 5% CO_2_ for 24 h. Following a 12 h starvation treatment with serum-free medium, the MAC-T cells were treated with valine-containing medium at concentrations of 0× Val, 1× Val, 2× Val, 4× Val, 8× Val, and PC (10% FBS) for an additional 12 h. The medium was then discarded, and each well was washed with pre-cooled PBS to remove residual medium. Next, 1 mL of pre-cooled TrizolTM reagent (Cat No. 15596026CN, Thermo Scientific, Waltham, MA, USA) was added to each well. After incubating at room temperature for 5 min, the cells were lysed by repeated pipetting, and the lysate was transferred to 1.5 mL RNase-free centrifuge tubes (Cat No. EP-150X-J, Wuhan Savel Biotechnology Co., Ltd., Wuhan, China). To each tube, 200 µL of pre-cooled chloroform (analytical grade) was added, followed by vigorous vortexing for 15 s using a vortex mixer (Cat No. SMV-3500, Wuhan Savel Biotechnology Co., Ltd., Wuhan, China). The samples were then centrifuged at 4 °C at 12,000× *g* for 15 min. Subsequently, 500 µL of the upper aqueous phase was transferred to new 1.5 mL RNase-free centrifuge tubes, and 500 µL of pre-cooled isopropanol was added. The mixture was thoroughly mixed and placed in the refrigerator for 10 min. After a second centrifugation at 4 °C at 12,000× *g* for 10 min, the supernatant was discarded. The RNA pellet was washed with 1 mL of pre-cooled 75% ethanol and subjected to a third round of centrifugation at 4 °C at 7500 rpm for 5 min. The ethanol was discarded, and once the RNA appeared transparent, 10–15 µL of DEPC water (Cat No. R0022, Shanghai Beyotime Biotechnology Co., Ltd., Shanghai, China) was added to dissolve the RNA. A 2 µL aliquot of the RNA solution was measured using a NanoDrop 2000 UV spectrophotometer (Cat No. NanoDrop2000, Thermo Scientific, Waltham, MA, USA) to determine the RNA concentration at 260 nm.

The PrimeScriptTM RT reagent Kit (TaKaRa) (Cat No. RR037A, TaKaRa Bio Inc., Kusatsu City, Shiga Prefecture, Japan) was used according to the manufacturer’s instructions to reverse-transcribe the RNA into cDNA using a gradient PCR thermal cycler (Cat No. SCI-G1000, Shanghai LanJieKe Technology Co., Ltd., Shanghai, China). Real-time fluorescent quantitative PCR (RT-qPCR) was performed using the TB Green Premix Ex TaqTM kit (Cat No. RR420A, TaKaRa Bio Inc., Shiga Prefecture, Japan) on a CFX Connect Real-Time PCR Detection System (Cat No. 17005940, Bio-Rad Laboratories, Inc., Hercules, CA, USA) to measure the expression levels of the α-casein-encoding gene and key factors in the mTOR signaling pathway, as detailed in Table 2. β-actin served as the internal reference gene, and the relative expression levels of the target genes were calculated using the 2-ΔΔCt method. The PCR cycling conditions were as follows: ① 95 °C for 30 s (one cycle); ② 95 °C for 5 s, 55 °C for 30 s, and 72 °C for 30 s (40 cycles); ③ 95 °C for 10 s, 65 °C for 5 s, and 95 °C for 5 s (one cycle).

#### 4.4.3. Determination of Phosphorylation of Related Proteins

The cell culture protocol for the Western blot analysis was identical to that used in the RT-qPCR experiment of this study. After treating MAC-T cells with amino acids, rapamycin, and 10% FBS for 12 h, the medium in the 6-well plates was discarded. Each well was washed 2–3 times with pre-cooled PBS to remove residual medium. Subsequently, 100 μL of RIPA lysis buffer (Cat No. AS1004, Pharmacare Holdings Limited, Johannesburg, South Africa), supplemented with 1% phosphatase inhibitor (Cat No. AS1008, Pharmacare Holdings Limited, Johannesburg, South Africa) and 1% protease inhibitor (Cat No. 04693159001, Roche Holding AG, Basel, Switzerland), was added to each well and incubated for 3–5 min to lyse the cells. The lysates were collected into 1.5 mL centrifuge tubes using a cell scraper. After a 30 min incubation on ice, the samples were centrifuged at 12,000 rpm for 5 min at 4 °C in a low-temperature high-speed centrifuge, and the supernatants, representing the total protein solution, were collected. The protein concentration of each samplhe from the difhferent treatment groups was determined using a BCA protein quantification kit (Cat No. AS1086, Pharmacare Holdings Limited, Johannesburg, South Africa), and the samples were adjusted to a uniform concentration. Equal amounts (40 μg) of protein were mixed with 5× protein loading buffer at a volume ratio of 4:1, boiled at 100 °C for 5 min, and prepared for electrophoresis. The proteins were subjected to SDS-PAGE electrophoresis and then transferred to a PVDF membrane (Cat No. IPVH00010, Millipore Corporation, Bedford, MA, USA). The membrane was blocked with blocking buffer (Cat No. P0023B, Shanghai Beyotime Biotechnology Co., Ltd., Shanghai, China) at room temperature for 1 h. After removing the blocking buffer, diluted primary antibodies were added, and the membrane was incubated overnight at 4 °C. The next day, the PVDF membrane was washed three times with TBST (prepared by mixing 1000 mL of TBS solution (Cat No. AS1024, Pharmacare Holdings Limited, Johannesburg, South Africa) with 1 mL of Tween-20 solution (Cat No. AS1100, Pharmacare Holdings Limited, Johannesburg, South Africa)) for 5 min each time. Secondary antibodies were then added, and the membrane was incubated at room temperature for 30 min, followed by four washes with TBST on a shaking platform at room temperature. Finally, the ECL chemiluminescent detection kit (Cat No. AS1059, Pharmacare Holdings Limited, Johannesburg, South Africa) was used to prepare a fresh ECL mixture (A:B = 1:1), which was applied to the protein side of the membrane. The membrane was exposed and developed in a darkroom. The films were scanned and archived, and the optical density values of the target bands were analyzed using the AlphaEaseFC software version 4.0 processing system. Detailed information about these antibodies is presented in Table 3.

### 4.5. Statistical Analysis

All data were calculated and organized using Excel 2016. Variance analysis was performed using the ANOVA function in SPSS 19.0 software, and multiple comparisons of means were conducted using Duncan’s test. Differences were considered statistically significant at *p* < 0.05 and highly significant at *p* < 0.01. Results are presented as the mean ± standard error.

## 5. Conclusions

The optimal addition range of valine for promoting α-casein synthesis in MAC-T cells is from 1× Val (6.384 mM) to 4× Val (25.536 mM). Valine exhibits a dose-dependent effect on enhancing α-casein synthesis, upregulating the expression of mTOR signaling pathway-related genes, and promoting the phosphorylation levels of associated proteins. Specifically, when the valine concentration reaches 4× Val (25.536 mM), the upregulation of mTOR signaling pathway-related gene expression and the promotion of protein phosphorylation are most pronounced. The inhibition of mTOR phosphorylation further underscores the critical role of the mTOR signaling pathway in valine-induced α-casein synthesis in MAC-T cells. In future experimental analyses, we will employ advanced statistical methods, including multiple regression analysis, to thoroughly investigate the interactions between valine concentration and other variables. This approach aims to provide more comprehensive theoretical support and practical guidance for the field of animal husbandry.

## Figures and Tables

**Table 1:** Levels of valine, rapamycin and fetal bovine serum added to the experimental culture medium.

Items	Groups										
	0× Val	0.25× Val	0.5× Val	1× Val	2× Val	4× Val	8× Val	PC	Rapamycin	Rapamycin + Val	Val
Valine (mM)	0.000	1.596	3.192	6.384	12.768	25.536	51.072	0.000	0.000	25.536	25.536
Rapamycin (mM)	0.000	0.000	0.000	0.000	0.000	0.000	0.000	0.000	100.000	100.000	0.000
Fetal Bovine Serum (%)	0.000	0.000	0.000	0.000	0.000	0.000	0.000	10.000	0.000	0.000	0.000

**Table 2:** Primer sequences of internal reference genes and target genes.

Genes	Gene Accession Number	Primer Sequence
*mTOR*	XM_001788228.1	F: ATGCTGTCCCTGGTCCTTATG
		R: GGGTCAGAGAGTGGCCTTCAA
*MLST8*	NM_001035411	F: ATCTGTACGCGAACTGTGCAG
		R: CATACATGCGAATGTGCTGG
*RPTOR*	NM_001192130	F: GTTTGGAATGCTGGATTGATC
		R: CTGAGTGTAGTTCTTGTTGAAGACC
*RRAGA*	NM_001035499	F: GAGGTTCTGATTTATGTGTTCGAC
		R: ACAATACTGGACCAGGCTTTGTAG
*RRAGB*	NM_001075279	F: GGGTAAGACCAGCATGAGGTCT
		R: CACAGTCCCACAGGTTCAATACC
*RRAGC*	NM_001076456	F: CATCCAGAAGGTGGTGTTTCAT
		R: GCATCAATGACATATATCAATGCTC
*RRAGD*	NM_001192828	F: CAGAGGTAAAGCCGAGGATCC
		R: TCCAAGAACAGAGTTTCGTTGG
*S6K1*	NM_205816.1	F: CTGGGGAAGAGGTGCTTCAG
		R: GTGCTCTGGTCGTTTGGAGA
*EIF4EBP1*	BC120290.1	F: GAACTCACCTGTGACCAAGA
		R: CTCAAACTGTGACTCTTCACC
*EIF4E*	NM_174310.1	F: ACGAAGTGACCTCGATCGTT
		R: AGTAGCTGTGTCTGCATGGG
*EEF2*	NW_618521.1	F: CTCTACCAAACCTTCCAGCG
		R: GCTGTTGGCTGACTTGCTGA
*RPS6*	NM_001010.2	F: AAGAGCTAGCAGAATCCGCA
		R: CGTGGAGTAACAAGACGCTG
*CSN1S1*	NM_181029.2	F: TCAACCCAGCTTGCTGCTTCTTCC
		R: GCCTAGCAAGAGCAACAGCCACAA
*CSN1S2*	NM_174528.2	F: AGCAGCTCTCCACCAGTGAGGAAA
		R: TGGGGCAAGGCGAATTTCTGGT
*β-actin*	NM_173979.3	F: GTCATCACCATCGGCAATGAG
		R: AATGCCGCAGGATTCCATG

**Table 3:** Detailed information of antibodies.

Primary Antibody	Antibody	Source	Catalog Number	Dilution Method	Dilution Ratio
	β-Actin	Tian De Yue	TDY051	5% fat-free milk	1:10,000
	P-mTOR	bioss	bs-3494R	5% egg white	1:500
	P-S6K1	affbiotech	AF3228	5% egg white	1:1000
	S6K1	affbiotech	AF6226	5% egg white	1:2000
	P-4EBP1	affbiotech	AF3432	5% egg white	1:1000
	4EBP1	affbiotech	AF6432	5% egg white	1:2000
	P-RPS6	affbiotech	AF3354	5% egg white	1:500
	RPS6	biorbyt	orb585017	5% egg white	1:1000
	P-eFF2	affbiotech	AF7220	5% egg white	1:500
	eEF2	biorbyt	orb584002	5% egg white	1:1000
Secondary antibody	HRP-Goat anti Rabbit	ASPEN	AS1107	5% fat-free milk	1:10,000
	HRP-Goat anti Mouse	ASPEN	AS1106	5% fat-free milk	1:10,000
	HRP-Rabbit anti Goat	ASPEN	AS1108	5% fat-free milk	1:10,000
	HRP-Goat anti Rat	ASPEN	AS1093	5% fat-free milk	1:10,000
	HRP-Rabbit anti Sheep	ASPEN	AS1245	5% fat-free milk	1:10,000

## Data Availability

I have uploaded the detailed data of this article to the following link: 10.6084/m9.figshare.28643597.

## Abbreviations

The following abbreviations are used in this manuscript:

MAC-T cells	Bovine mammary epithelial cells line
BCAA	Branched-chain amino acids
RT-qPCR	Real-time quantitative PCR
Val	Valine
FBS	Fetal bovine serum
10%FBS	10% fetal bovine serum
ELISA	Enzyme-linked immunosorbent assay
mTOR	Mammalian target of rapamycin
S6K1	Ribosomal protein S6 kinase 1
4EBP1	Eukaryotic initiation factor 4E-binding protein 1
RPS6	Ribosomal protein S6
eEF2	Eukaryotic translation elongation factor 2
*EIF4E*	*Eukaryotic initiation factor 4E*
*EEF2*	Eukaryotic elongation factor 2
BMECs	Bovine mammary epithelial cells
RPS6	Ribosomal *protein S6*
